# Quality Improvement in Pediatric Head Trauma with PECARN Rules Implementation as Computerized Decision Support

**DOI:** 10.1097/pq9.0000000000000019

**Published:** 2017-05-16

**Authors:** Shireen M. Atabaki, Brian R. Jacobs, Kathleen M. Brown, Samira Shahzeidi, Nia J. Heard-Garris, Meghan B. Chamberlain, Robert M. Grell, James M. Chamberlain

**Affiliations:** From the * Children’s National Medical Center, Washington, D.C.; † George Washington University School of Medicine and Health Sciences, Washington, D.C.; and ‡ University of Michigan, Ann Arbor, Mich.

## Abstract

**Background:**

For the 1.4 million emergency department (ED) visits for traumatic brain injury (TBI) annually in the United States, computed tomography (CT) may be over utilized. The Pediatric Emergency Care Applied Research Network developed 2 prediction rules to identify children at very low risk of clinically important TBI. We implemented these prediction rules as decision support within our electronic health record (EHR) to reduce CT.

**Objective:**

To test EHR decision support implementation in reducing CT rates for head trauma at 2 pediatric EDs.

**Methods:**

We compared monthly CT rates 1 year before [preimplementation (PRE)] and 1 year after [postimplementation (POST)] decision support implementation. The primary outcome was change in CT use rate over time, measured using statistical process control charts. Secondary analyses included multivariate comparisons of PRE to POST. Balancing measures included ED length of stay and returns within 7 days after ED release.

**Results:**

There were 2,878 patients with head trauma (1,329 PRE and 1,549 POST) included. Statistical process control charts confirmed decreased CT rates over time POST that was not present PRE. Secondary statistical analyses confirmed that CT scan utilization rates decreased from 26.8% to 18.9% (unadjusted Odds Ratio [OR], 0.64; 95% Confidence Interval [CI], 0.53 -0.76; adjusted OR, 0.71; 95% CI, 0.58 -0.86). Length of stay was unchanged. There was no increase in returns within 7 days and no significant missed diagnoses.

**Conclusions:**

Implementation of EHR-integrated decision support for children with head trauma presenting to the ED is associated with a decrease in CT utilization and no increase in significant safety events.

## Abstract 2

Supplemental Digital Content is available in the text.

## INTRODUCTION

In the United States, there are approximately 1.4 million patients with traumatic brain injury (TBI) treated in emergency departments (EDs) annually, with close to 500,000 TBI-related visits among children under 15 years of age.^1^ Computed tomography (CT) is an important tool in the evaluation of patients with head trauma, allowing early identification of life-threatening intracranial hemorrhage; however, increasing evidence suggests that CT is overused. Eighty-eight percentage to 92% of patients with head trauma have mild TBI and the rate of positive CT scan demonstrating any intracranial injury in this setting is less than 10%.^2^ The rate of clinically significant intracranial injury (ie, requiring surgical intervention) is much lower.^3^ There is considerable practice variation among emergency providers in CT use for patients with mild TBI.^4–6^ Published guidelines for CT use in the setting of mild TBI have recommended CT scans for minor symptoms such as vomiting.^7,8^ Between 1995 and 2003, the United States witnessed a near doubling of pediatric cranial CT.^9^

CT is not without associated costs and risks. Radiation attributable cancer mortality risk from exposure to cranial CT in childhood is estimated to be as high as 1 in 1,400.^10^ Cranial CT scans performed in the first 22 years of life may triple the risk of leukemia or brain tumors.^11^ To address these concerns, the National Cancer Institute and the Food and Drug Administration have recommended a decrease in radiation exposure by eliminating unnecessary CT scans, with special emphasis on the pediatric population.^12,13^

Several studies have derived prediction rules to assist emergency providers with decision making for obtaining CT scans for children with head trauma.^14–17^ These early decision rules had relatively small sample sizes and most lacked prospective validation.^14–16^ In 2009, the Pediatric Emergency Care Applied Research Network (PECARN)^18^ developed and validated 2 prediction rules to identify those children at very low risk of clinically important traumatic brain injury (ciTBI) after head trauma; 1 for children younger than 2 years and 1 for children 2 years and older.^19^ The rule for children younger than 2 years included 6 predictors: altered mental status, nonfrontal scalp hematoma, loss of consciousness for 5 seconds or more, severe mechanism of injury, palpable skull fracture, or not acting normally according to the parent.^19^ The rule for children 2 years and older included 6 predictors: abnormal mental status, any loss of consciousness, history of vomiting, severe injury mechanism, clinical signs of basilar skull fracture, or severe headache.^19^ These prediction rules were derived and validated in very large patient cohorts and have excellent performance characteristics.^19,20^ Neither PECARN prediction rule missed patients with need for neurosurgical intervention in the validation populations.

We performed this study to test the implementation of the PECARN prediction rules in clinical practice using the electronic health record (EHR). We hypothesized that the implementation of the PECARN prediction rules in the EHR would be associated with a decrease in the rate of cranial CT for head trauma.

## METHODS

### Study Design

We performed a time-series trial to compare rates of CT before and after implementation of a quality improvement project of workflow-integrated decision support. This quality improvement project focused on evidence-based use of cranial CT for head trauma with the goal of reduction of unnecessary CT scans. This study met our Institutional Review Board criteria for a quality improvement study, for which Institutional Review Board approval is not required.

### Study Setting and Population

The setting was the ED of a Level-1 trauma center at a children’s hospital and a pediatric ED within a community hospital, with a combined annual patient volume of approximately 108,000 visits per year. Both EDs are staffed by the same physicians and use the same EHR. We reviewed ED visits between January 1, 2010, and March 31, 2012, and included all patients from birth to 18 years of age with diagnoses indicative of head trauma. We excluded patients with trivial injury (lacerations and abrasions) and patients who were transferred after receiving an evaluation for head trauma at another hospital.

### Existing Standard of Care

There were no significant changes in staff during the study period. Annual staff turnover constitutes less than 10% of our overall ED faculty and fellows. Final decisions on CT scan for head trauma are always made in consultation with ED faculty or fellows in instances where trainees (medical students and residents) or physician extenders (physician assistants) are involved. There were no differences in triage policies or facility changes, such as different access to CT, during the study period.

**Fig. 1.**
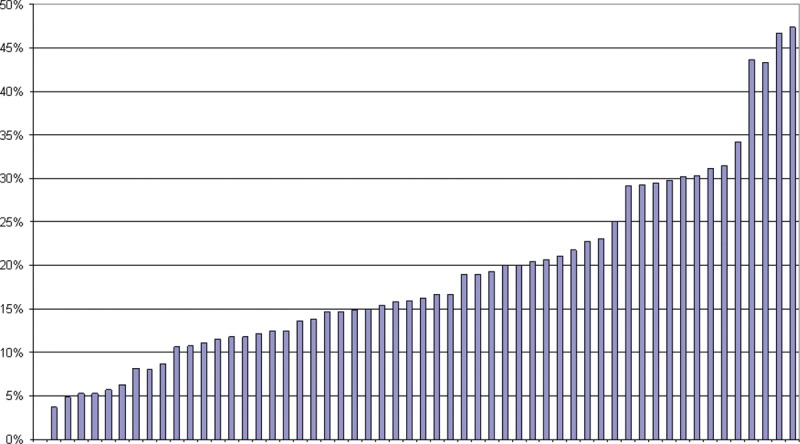
Proportion of head injury patients with head CT ordered by individual EPs during the period from January 2010 to March 2011. Benchmarking feedback sent quarterly to all EPs with their unique identifier. Percentage of head CT orders are shown on the *y* axis, and anonymous individual providers are shown on the *x* axis. EP, emergency provider.

Rates of CT use have increased over the last 2 decades.^9^ Physicians have traditionally ordered CT scans based on clinical judgment and without guidance from a clinical decision rule. This has resulted in an approximately 10-fold difference in CT ordering rates at our EDs (Fig. 1). Despite the publication of the National Cancer Institute and Food and Drug Administration warnings for judicious CT use in pediatrics in 2002,^11,12^ and publication of the evidence-based PECARN decision rules in 2009,^18^ CT use had not decreased at our institution.

### Quality Improvement Strategy

#### Planning the Intervention.

Our hospital adopted computerized provider order entry in December 2005 and a full EHR in May 2008. We performed a quality improvement intervention to reduce unnecessary CT scans for mild TBI in our EDs. The intervention in this study was the incorporation of the PECARN prediction rules into the EHR of both EDs. A multidisciplinary team of emergency physicians, database analysts, and nursing leadership designed and implemented real-time decision support into the EHR. The decision support was embedded into a new order: “Trauma Head CT.” The decision tool form requires the user to input data for 6 fields. Conditional logic, based on the risk stratification algorithm from the PECARN study, displays the risk stratum for the patient: low risk—CT is not recommended; high risk—CT is recommended; and intermediate risk—consider CT or observation (**Supplemental Digital Content 1**, http://links.lww.com/PQ9/A5). Use of the decision rule was encouraged but not required. Normal workflow required completion of the decision rule when ordering a “Trauma Head CT,” but providers were able to order a “Head CT” and bypass the decision rule. Providers also could access the decision rule independently of computerized provider order entry as a stand-alone form.

At rule implementation, we provided hospital-wide education to all emergency providers, including those based in the ED and rotating trainees. We provided education on the electronic decision support through lectures, discussions at medical staff meetings, and e-mail reminders (**Supplemental Digital Content 2**, http://links.lww.com/PQ9/A6). Educational reference included laminated pocket cards, posters and flyers, and e-mail. Providers were encouraged to use the tool when considering ordering cranial CT for suspected TBI and when assessing any patient with head trauma.

### Planning the Study of the Intervention

We used standard improvement science techniques to study the effects of this decision support intervention. These included time-series analyses, and statistical process control charts, to study the change in rate of CT scans over time. Additionally, we used traditional multivariable statistical techniques to compare CT scan rates before and after the implementation. We planned to use 12 months to establish baseline CT rates before the intervention, followed by 12 months of data collection after the intervention. We performed the intervention more than a year after the publication of the PECARN decision rule paper^19^ and nearly 2 years after the PECARN decision rule was presented as an abstract^21^ to allow natural adoption into clinical practice before the EHR intervention.

### Provider Feedback

Throughout this quality improvement initiative, providers at both EDs received feedback on CT reduction rates from the division chief (J. M. C.) and the project principal investigator (S. M. A.). The division chief also distributed information on preimplementation (PRE) variation in CT ordering rates by provider for both EDs (Fig. 1).

### Methods of Evaluation

We recorded monthly rates of CT use and retrospectively reviewed charts for all patients who met eligibility criteria. Eligible subjects included patients aged 0–18 years of age presenting to our ED with a complaint of head trauma between January 1, 2010, and March 31, 2012.

The implementation period included the months of January to March 2011. Thus, the PRE phase was from January 1, 2010, to December 31, 2010, and the postimplementation (POST) phase was from April 1, 2011, to March 31, 2012.

We compared CT scan rates and the proportion of positive scans of the PRE phase to the POST phase. Cranial CT was considered positive in the presence of any of the following: intracranial hemorrhage or contusion, cerebral edema, or skull fracture. Glasgow Coma Scale scores were recorded; when absent in the EHR, we looked for other indicators of normal mental status, such as text that stated “Alert and appropriate.” When such descriptors were present, we considered these patients equivalent to a Glasgow Coma Scale of 15. Safety events were defined as return visits within 7 days and a missed diagnosis of ciTBI in cases of return visits to the ED.

### Analysis

The primary outcome was the change in monthly rate of CT ordering after implementation of the decision support intervention. Secondary outcomes included the proportion of patients with the decision support form completed and the proportion of positive CTs. Balancing measures, other indices of quality that might be affected by this intervention, included ED length of stay and the rate of return to the hospital within 7 days for reevaluation of head injury. Because our hospital has the only pediatric intensive care unit in the city, any serious missed injuries would be detected by readmission.

Our primary statistical analysis was time-series analysis using statistical process control charts. Control charts were introduced by Shewhart^22^ in the 1920s as an intuitive, graphical method for determining statistically significant changes in rates or events over time. Statistical process control charts are favored over simple comparisons of PRE versus POST because they provide important information about the effects of time. In addition, we performed traditional multivariate statistical testing to compare the probability of ordering a CT based on the presence of the decision support tool (ie, PRE versus POST), while controlling for other clinical variables. We used a *P* value of less than 0.05 as the threshold for statistical significance for all testing. We used SPSS (version 20, IBM, Armonk, N.Y.) for all analyses.

## RESULTS

### Patient Characteristics

Overall, there were 2,878 patients with head trauma; there were 1,329 PRE patients and 1,549 POST patients. The decision support was utilized and completed for 387/1,549 (24.9%) of POST patients. Table 1 depicts the characteristics of the study patients.

**Table 1.**
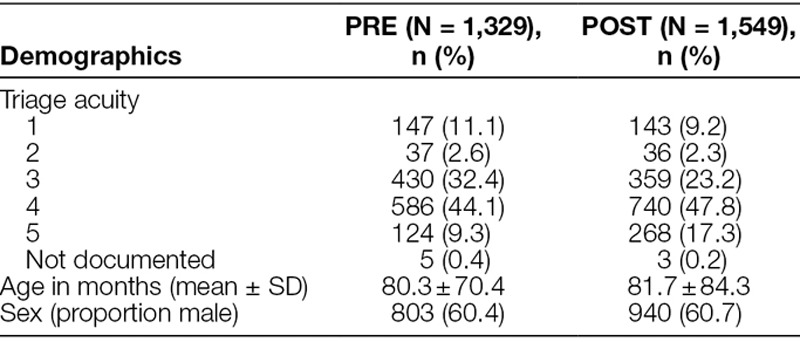
Demographic Data, PRE of Decision Support, and POST

### Primary Outcome

**Fig. 2.**
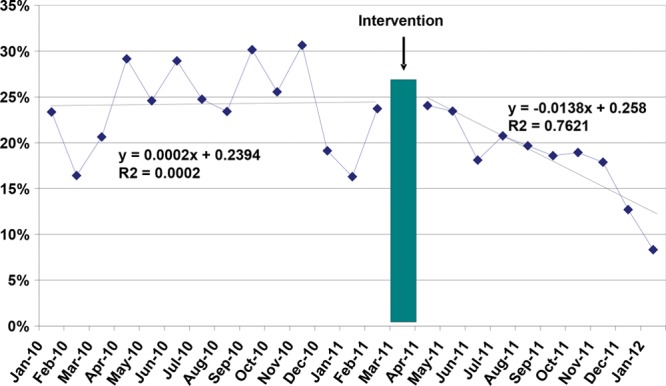
Proportion of blunt head trauma patients with CT performed. Percentage of head CTs performed is shown on the *y* axis, and data before and after the intervention by month is shown on the *x* axis. Dotted line represents regression line for PRE and POST.

**Fig. 3.**
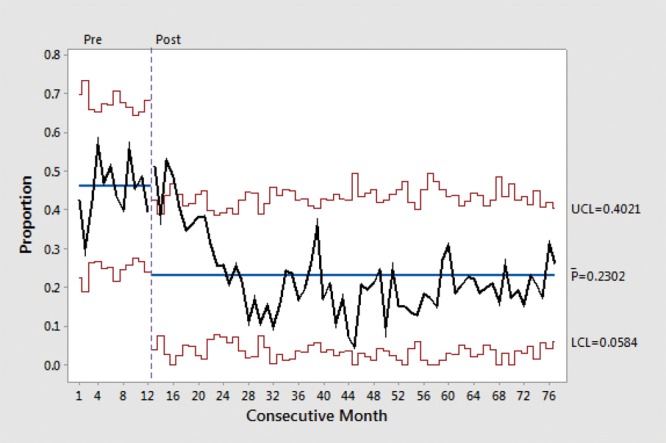
Proportion of blunt head trauma patients with CT performed over an extended 80-month period (note that months 13–15 are omitted during the transition period). Percentage of head CTs performed is shown on the *y* axis, and data before and after the intervention by month is shown on the *x* axis. LCL, lower control limit; UCL, upper control limit.

Figure 2 depicts the change in CT ordering rates over time. There was a significant decrease in CT ordering after the intervention; the last 3 months meet several statistical criteria for a significant change from baseline. Figure 2 also depicts the regression line for PRE and POST; the line is flat before the intervention and has a negative slope after the intervention. Figure 3 demonstrates that this negative slope after intervention was sustained over an extended 5-year period. Overall, there were 648 patients with CT scans and 2,230 without, 356 of 1,329 patients (27%) in the PRE phase and 292 of 1,549 (19%) in the POST phase. Multivariate analyses controlling for sex and triage acuity confirmed the association of POST with decreased risk of CT (adjusted odds ratio [OR] = 0.63; 95% confidence interval [CI], 0.52–0.76; Table 2). Age was not statistically associated with rate of CT ordering.

**Table 2.**
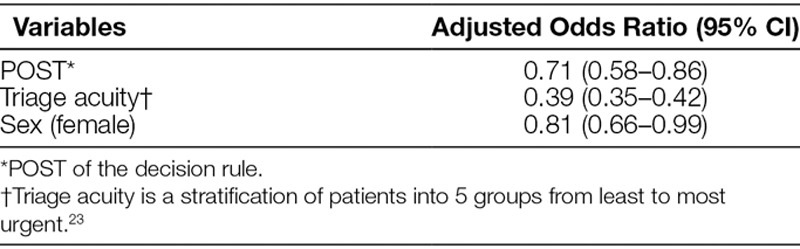
Results of Multivariable Analysis Controlling for Sex and Triage Acuity

In addition to performing multivariable analyses, which control for severity level, we also performed the analyses after excluding triage levels 4 and 5 (the lowest acuity levels).^23^ Excluding triage levels 4 and 5, the CT rate decreased from 46% to 30%, and the slope of the change was -0.018, which is steeper than the slope for all patients (-0.11).

### Secondary Outcomes

The proportion of positive CTs was 90/356 (25.3%) PRE and 68/292 (23.2%) POST (*P* NS). The mean length of stay was 198 minutes in both groups (*P* = 0.93, Student’s *t* test). There were no significant safety events. There was no significant change in the rate of return visits to the ED within 7 days (10/1,329 PRE versus 14/1,549 POST (OR, 1.20; 95% CI, 0.53–2.72). None of these returns was associated with a missed diagnosis.

## DISCUSSION

Our results demonstrate a strong temporal association between EHR workflow-integrated evidence-based decision support and reduction in head CT rates for children presenting to the ED with head trauma. The decrease in CT rate is consistent with the gradual uptake of evidence-based practice by medical providers over time. The average rate of head CT ordering changed from 27% to 19% comparing the entire PRE period to the entire POST period, but the actual decrease in monthly rates was much greater, decreasing from 26% to 13% after implementation (Fig. 2). There were no significant safety events (ie, significant missed TBI resulting in ICU admission or death) associated with this change; specifically, there was no change in the rate of return visits to the ED within 7 days and in cases of return visits to the ED, none were associated with a missed diagnosis. Furthermore, there was no associated increase in length of stay after implementation, which might be expected if more patients were being observed in the ED rather than having a CT.

Before implementation of this quality improvement project in our 2 EDs, many emergency providers failed to change their behavior, despite the fact that the PECARN prediction rules were published in 2009 and the results were widely disseminated. We feel comfortable that the reduction in CT rates is not simply a result of the publication of the clinical decision rules, rather it was related to their implementation as real-time decision support. These findings are consistent with prior research demonstrating that publication of evidence alone is not enough to change practice.^24^ In addition, a single-site study of implementation of the PECARN rules starting in 2010 demonstrated a 10% reduction in CT use.^25^ Adoption of the EHR has improved medication safety^26^ and adherence to clinical guidelines for screening and diagnostic testing, especially in the ambulatory setting.^27^ In our study, implementation of the PECARN decision rules into the EHR and normal workflow led to almost immediate cranial CT reduction for children with head trauma. As more hospitals adopt the EHR, incorporation of decision support into the EHR can be leveraged to provide rapid dissemination and reduce the traditional 13-year lag for new knowledge implementation.^28^

A systematic review demonstrated that computerized clinical decision support systems improve quality of care using process measures, but the effect on patient outcomes is inconsistent across studies.^29^ Our study used a process measure, the performance of CT scan. However, this process measure is tightly linked with a reduction of exposure to ionizing radiation, which could affect patient outcomes, especially if similar results are achieved nationally.

### Proposed Reasons for Benefit

A systematic review of 70 studies using clinical decision support to improve clinical practice identified 4 features critical to success: automatic provision of decision support as part of workflow, provision of recommendations rather than assessments, provision of decision support at the time of decision making, and computer-based decision support.^30^ Our study was successful because our implementation of decision support incorporated all 4 features. Our decision support tool was designed by physicians and nurses and incorporated as computer-based decision support into the course of normal EHR workflow.^31^ We provided decision support at the time of decision making and made this flexible; providers could access the decision tool before ordering CT or were prompted to complete the tool at the time of CT ordering.

We provided access to the evidence underlying the decision rules within the computerized decision support. Emergency providers seek to maximize sensitivity to avoid missed diagnoses and may order diagnostic tests to reduce the potential for litigation. Providing evidence-based real-time access to risk stratification helps support their decision to forgo CT scan when risk is extremely low. Once completed, these decision support tools become part of the medical record and provide documentation for decision making.

Emergency physicians support development and use of clinical decision rules^31^ but may apply rules incorrectly without associated decision support.^32,33^ Of note, US physicians have been less likely than physicians from other countries to adopt the use of specific published clinical decision rules, despite similar rates of awareness.^33^ As more hospitals adopt EHRs, embedded decision support is a natural next step, but software designers will need to work with medical providers to ensure usability and integration into normal workflow.

### Limitations

This study had several limitations. First, we did not perform a randomized controlled trial and we can report only on an association between outcomes and the intervention. However, the association was strong and the effect was temporally related to the intervention. There was not an effect before the intervention, despite presentations of the PECARN decision rules, nationally and locally, beginning 2 years before tool implementation. Second, use of the decision tool was not mandatory. The tool was designed as a required form when ordering a “Trauma Head CT;” however, the form had to be independently accessed by the provider if a CT scan was not ordered. Furthermore, because ED orders are shared with inpatient settings, medical providers were able to bypass the clinical decision support tool by ordering a “Head CT” rather than a “Trauma Head CT;” this may have biased our results against demonstrating an effect of the intervention. Third, interviews with staff indicate that some providers became very familiar with the PECARN decision rules and no longer needed to use the computerized process; we are unable to measure the magnitude of this effect. These interviews suggest that the decision support was helpful as a reference. Finally, this study was performed at 2 hospitals. Adoption of decision rules is affected by local practice and culture and similar results may not be achieved in different settings.^33^

### Implications for Future Research

Since the study, we have initiated periodic audit and feedback to our emergency providers, with benchmarking data comparing their rate of CT for head trauma to their peers (Fig. 1). We will measure the effects of this feedback. Surveys of emergency providers about reasons for failing to adhere to decision rule recommendations may focus future interventions.^31^ Incorporation of additional reference literature, including management strategies for intermediate-risk groups with observation in lieu of cranial CT, may lead to further reduction of cranial CT and should be studied.^34^

## CONCLUSIONS

We have demonstrated that implementation of decision support in the EHR is associated with a decrease in the rate of cranial CT for pediatric head trauma without an increase in missed ciTBI. Implementation of the PECARN clinical decision rules, which categorize children with head trauma into low, intermediate, and high risk of ciTBI into ED workflow has the potential to safely decrease unnecessary cranial CT. Widely adopted with improved workflow implementation, this strategy could lead to a national reduction of unnecessary CT scans for children with head trauma and reduce the burden of radiation exposure for children.

## DISCLOSURE

The authors have no financial interest to declare in relation to the content of this article.

## References

[1] Faul M Xu L Wald MM Traumatic Brain Injury in the United States: Emergency Department Visits, Hospitalizations and Deaths 2002–2006. 2010 Atlanta, Ga.: Centers for Disease Control and Prevention, National Center for Injury Prevention and Control.

[2] Davis RL Mullen N Makela M Cranial computed tomography scans in children after minimal head injury with loss of consciousness. Ann Emerg Med. 1994;24:640 645. 8092590 10.1016/s0196-0644(94)70273-x

[3] Schunk JE Rodgerson JD Woodward GA The utility of head computed tomographic scanning in pediatric patients with normal neurologic examination in the emergency department. Pediatr Emerg Care. 1996;12:160 165. 8806136 10.1097/00006565-199606000-00004

[4] Quayle KS Jaffe DM Kuppermann N Diagnostic testing for acute head injury in children: when are head computed tomography and skull radiographs indicated? Pediatrics. 1997;99:E11. 10.1542/peds.99.5.e11 9113968

[5] Stanley RM Hoyle JD Jr Dayan PS; Pediatric Emergency Care Applied Research Network (PECARN). Emergency department practice variation in computed tomography use for children with minor blunt head trauma. J Pediatr. 2014;165:1201 1206.e2. 25294604 10.1016/j.jpeds.2014.08.008

[6] Hennes H Lee M Smith D Clinical predictors of severe head trauma in children. Am J Dis Child. 1988;142:1045 1047. 3177299 10.1001/archpedi.1988.02150100039021

[7] Homer CJ Kleinman L Technical report: minor head injury in children. Pediatrics. 1999;104:e78. 10586012 10.1542/peds.104.6.e78

[8] The management of minor closed head injury in children. Committee on Quality Improvement, American Academy of Pediatrics. Commission on Clinical Policies and Research, American Academy of Family Physicians. Pediatrics. 1999;104:1407 1415. 10585999

[9] Blackwell CD Gorelick M Holmes JF Pediatric head trauma: changes in use of computed tomography in emergency departments in the United States over time. Ann Emerg Med. 2007;49:320 324. 17145113 10.1016/j.annemergmed.2006.09.025

[10] Brenner D Elliston C Hall E Estimated risks of radiation-induced fatal cancer from pediatric CT. AJR Am J Roentgenol. 2001;176:289 296. 11159059 10.2214/ajr.176.2.1760289

[11] Pearce MS Salotti JA Little MP Radiation exposure from CT scans in childhood and subsequent risk of leukaemia and brain tumours: a retrospective cohort study. Lancet. 2012;380:499 505. 22681860 10.1016/S0140-6736(12)60815-0 PMC3418594

[12] Radiation risks and pediatric computed tomography (CT): a guide for health care providers. National Cancer Institute Web site. Available at http://cancer.gov/cancerinfo/causes/radiation-risks-pediatric-ct. Accessed September 13, 2016.

[13] Food and Drug Administration. FDA Public Health Notification. Pediatr Radiol. 2002;32:314 316. 11956716 10.1007/s00247-002-0687-6

[14] Palchak MJ Holmes JF Vance CW A decision rule for identifying children at low risk for brain injuries after blunt head trauma. Ann Emerg Med. 2003;42:492 506. 14520320 10.1067/s0196-0644(03)00425-6

[15] Atabaki SM Stiell IG Bazarian JJ A clinical decision rule for cranial computed tomography in minor pediatric head trauma. Arch Pediatr Adolesc Med. 2008;162:439 445. 18458190 10.1001/archpedi.162.5.439

[16] Oman JA Cooper RJ Holmes JF; NEXUS II Investigators. Performance of a decision rule to predict need for computed tomography among children with blunt head trauma. Pediatrics. 2006;117:e238 e246. 16418311 10.1542/peds.2005-1063

[17] Haydel MJ Shembekar AD Prediction of intracranial injury in children aged five years and older with loss of consciousness after minor head injury due to nontrivial mechanisms. Ann Emerg Med. 2003;42:507 514. 14520321 10.1067/s0196-0644(03)00512-2

[18] The Pediatric Emergency Care Applied Research Network. The Pediatric Emergency Care Applied Research Network (PECARN): rationale, development, and first steps. Pediatr Emerg Care 2003;19:185 193. 12813308 10.1097/01.pec.0000081245.98249.6e

[19] Kuppermann N Holmes JF Dayan PS; Pediatric Emergency Care Applied Research Network (PECARN). Identification of children at very low risk of clinically-important brain injuries after head trauma: a prospective cohort study. Lancet. 2009;374:1160 1170. 19758692 10.1016/S0140-6736(09)61558-0

[20] Pearce MS Patterns in paediatric CT use: an international and epidemiological perspective. J Med Imaging Radiat Oncol. 2011;55:107 109. 21501396 10.1111/j.1754-9485.2011.02240.x

[21] Kuppermann N Holmes JF Dayan PS Blunt head trauma in the Pediatric Emergency Care Applied Research Network (PECARN). [Abstract] E-PAS2007:615705.3 (Presented at the Annual Meeting of the Pediatric Academic Societies, Toronto, Ontario, May 2007, and the Annual Meeting of the Society for Academic Emergency Medicine, Chicago, IL, May 2007).

[22] Shewhart WA Finding causes of quality variations. Manufacturing Industry 1926;11:125 128.

[23] Baumann MR Strout TD Evaluation of the Emergency Severity Index (version 3) triage algorithm in pediatric patients. Acad Emerg Med. 2005;12:219 224. 15741584 10.1197/j.aem.2004.09.023

[24] Graham ID Stiell IG Laupacis A Emergency physicians’ attitudes toward and use of clinical decision rules for radiography. Acad Emerg Med. 1998;5:134 140. 9492134 10.1111/j.1553-2712.1998.tb02598.x

[25] Nigrovic LE Stack AM Mannix RC Quality improvement effort to reduce cranial CTs for children with minor blunt head trauma. Pediatrics. 2015;136:e227 e233. 26101363 10.1542/peds.2014-3588 PMC5660895

[26] Evans RS Pestotnik SL Classen DC A computer-assisted management program for antibiotics and other antiinfective agents. N Engl J Med. 1998;338:232 238. 9435330 10.1056/NEJM199801223380406

[27] Terrell KM Perkins AJ Dexter PR Computerized decision support to reduce potentially inappropriate prescribing to older emergency department patients: a randomized, controlled trial. J Am Geriatr Soc. 2009;57:1388 1394. 19549022 10.1111/j.1532-5415.2009.02352.x

[28] Zerhouni EA Clinical research at a crossroads: the NIH roadmap. J Investig Med. 2006;54:171 173. 10.2310/6650.2006.X0016 17152855

[29] Garg AX Adhikari NK McDonald H Effects of computerized clinical decision support systems on practitioner performance and patient outcomes: a systematic review. JAMA. 2005;293:1223 1238. 15755945 10.1001/jama.293.10.1223

[30] Kawamoto K Houlihan CA Balas EA Improving clinical practice using clinical decision support systems: a systematic review of trials to identify features critical to success. BMJ. 2005;330:765. 15767266 10.1136/bmj.38398.500764.8F PMC555881

[31] Trivedi MH Kern JK Marcee A Development and implementation of computerized clinical guidelines: barriers and solutions. Methods Inf Med. 2002;41:435 442. 12501817

[32] Graham ID Stiell IG Laupacis A Awareness and use of the Ottawa ankle and knee rules in 5 countries: can publication alone be enough to change practice? Ann Emerg Med. 2001;37:259 266. 11223761 10.1067/mem.2001.113506

[33] Brehaut JC Stiell IG Visentin L Clinical decision rules “in the real world”: how a widely disseminated rule is used in everyday practice. Acad Emerg Med. 2005;12:948 956. 16166599 10.1197/j.aem.2005.04.024

[34] Nigrovic LE Schunk JE Foerster A; Traumatic Brain Injury Group for the Pediatric Emergency Care Applied Research Network. The effect of observation on cranial computed tomography utilization for children after blunt head trauma. Pediatrics. 2011;127:1067 1073. 21555498 10.1542/peds.2010-3373

